# A novel measuring chamber and automation platform for mammalian cell culture processes

**DOI:** 10.1186/1753-6561-9-S9-P30

**Published:** 2015-12-14

**Authors:** Tim H Lücking, Christoph Busse, Christian Lüder, David Bulnes-Abundis, Dörte Solle, Thomas Scheper

**Affiliations:** 1 Institute of Technical Chemistry, Leibniz University Hannover, Callinstr. 5, D-30167 Hannover, Germany

## Background

One of the biggest challenges of biotechnological production is the implementation of Process Analytical Technology (PAT) to obtain a highly automated, monitored and controlled process. Therefore the integration of suitable analytical sensors has to be faced at the earliest opportunity during process development. But sensor implementation for glass or disposable reactors encounters different issues. These systems are not designed to support well-established and standardized analytic ports for more complex probes used for stainless steel reactors. The here presented measuring chamber, combined with an automation platform enable an accelerated integration of PAT during all stages of process development for any reactor type.

## Development of a bypass measuring chamber

The measuring chamber was built according to concurrent sterile construction guidelines to achieve a design which does not compromise the sterility of a cell culture process. A modular design was chosen, thus it is possible to maximize the flexibility and adapt the chamber onto different kind of probe dimensions and measurement principles.

One of the main objectives was to develop a bypass measuring chamber that enabled the use of standard sensors, without the need of reconstructing the probes itself. Larger stainless steel bioreactors, as used in production scale, provide mainly two different ports for mounting analytical probes or other peripheral devices for inline measurements. Those are the Ingold-port, a mostly sideways accessible opening with a diameter of 25 mm and the primarily lid-mounted probes for PG 13.5-Ports, which have 12 mm in diameter.

**Figure 1:**
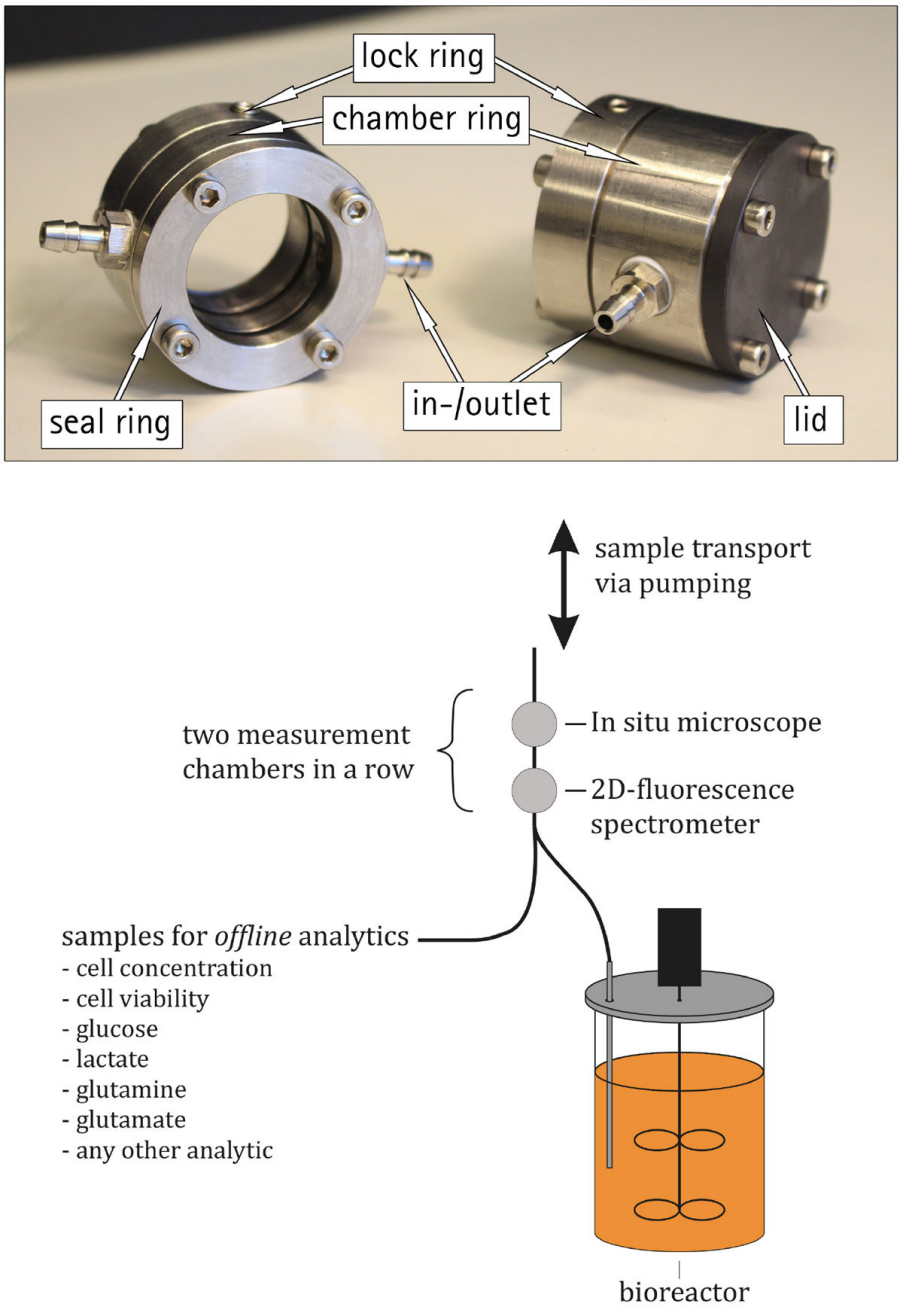
**Picture of the chambers for two different measurement principles (top) and schematic of measuring chamber setup (bottom)**.

At one end of every chamber a lock ring clamps onto the outer part of the probe and fixes the position of the chamber. The chamber ring has two opposing openings that are used as an inlet and outlet for the sample. The chamber is sealed against the bottom and top part with one O-ring on each side. The lid, which design depends on the dimensions of the probe and the underlying measuring principle, seals the chamber. Optical probes, e.g. the 2D-fluorescence spectrometer BioView® (DELTA, Hørsholm, Denmark), are measuring light reflection at the end of the probe [1,2]. Other systems like the BioPAT®Spectro NIR (Sartorius Stedim Biotech BmbH, Göttingen, Germany) or the in house developed insitu microscope (ISM) are measuring through a measuring gap [3-5]. Therefore a possibility to adapt the chamber to the respective analytic principle has to be available. This flexibility was achieved by the two lid-designs, a closed lid for terminal measurements and a seal ring for placing it close to a measurement gap. Figure 1 (top) shows the two versions.

The chamber is attached to the reactor via tubing to a sample taking port. Figure 1 (bottom) depicts a schematic of the setup, with the sample transport through measurement chambers and the possibility for *offline *sample taking after the bypass measurements. The chamber design allows the operation of different sensors in series. Thereby it is possible to connect more than one sensor into only one sample taking port. This makes it possible to test different analytical sensors during only one process in small scale.

## Measuring chamber analytics

During different cultivations in 2 L glass and 15 L stainless steel reactors, the 2D-fluorescence spectrometer and the in situ microscope were integrated into the bypass measuring chamber system. Especially in the 2 L glass reactor the measuring chambers show their capability. As mentioned before, most dedicated analytical sensors are designed for the Ingold port dimensions. The glass reactor only has PG 13.5 ports on the lid, thus the use of those sensors within this system was not possible up to now. But with the use of measuring chambers, a random number of all kinds of sensors can be included into any bioreactor concept.

The comparison of data acquired within the measurement chamber with data from an inline mounted spectrometer and in situ microscope has proven the comparability of the different measurement placements. The growth of CHO cells was not influenced by the transport to and through the chambers, no effect to the process was observed. In addition, the use of the novel measuring chambers enables more reproducible and robust measurements. This is due to the fact that optical measurements are often distorted by the process itself, e.g. reflection at the surface of air bubbles from the aeration or influences from different stirrer speeds. All these effects are not appearing inside the measuring chamber, whereby the measurements are decoupled from the reactor system. This enables the comparison between different scales and the transfer within the process up scale.

## Conclusions

The versatile measuring chambers enable the usage of well-established sensors within new or before unsuitable reactor and cultivation concepts. The comparison from spectra obtained within the chamber and the inline mounted sensor promise a data transfer during process scale up and thus enable the use of such a chamber within the early development of a process. The chamber enables the detachment form measurement systems and reactor systems.

This technology will enable new approaches during process development as a larger range of analytical tools can be tested in early development stages. In addition, the measuring chamber can be used for the integration within continuous manufacturing. Mounting different sensors inside or between up- and downstream processing steps and thus gaining more information and achieving a more automated concept, e.g. controlling the chromatographic purification via measuring the remaining impurities with spectroscopic sensors.
